# Some Scientific Aspects of Insanity

**Published:** 1891-12-05

**Authors:** 


### Some Scientific Aspects of Insanity.

### V.—CAUSES OF INSANITY.

Insanity is, as we have seen, a failure on the part of the nervous system to perform its function of adapting the individual to his surroundings. This failure of adaptation is generally due to three distinct causes.

1st. Arrested or imperfect development.

2nd. Overstrain from the pressure of adverse circumstances in the surroundings.

3rd. Overstrain from noxious changes within the organism.

It is well to remember that all these causes may operate together, or any two of them may be combined, or each of them may act singly in the causation of insanity. Indeed, it is seldom that one cause alone is sufficiently powerful. For instance, the strain that is strong enough to produce madness in one person may not affect to a serious extent a man of sterner fibre. Yet every individual has his or her breaking point, and if the strain is only sufficiently strong and sufficiently prolonged, the most perfect nervous system will ultimately give way.

I. Arrested Development.—The development of the nervous system may be wholly or partially arrested at any stage of its active growth. There may also be a flaw or a weakness due to a want of the sufficient developmental energy required to build up and complete the complicated structure in its entirety.

Insanity is not an inherited disease any more than consumption is, but there are certain tendencies towards instability of the nervous system along with peculiarities of disposition, which are hereditary. The great fundamental fact of insane heredity is the want of developmental energy handed down from parent to offspring. An individual's mental development may, through want of this inherent force, cease in early childhood, thus causing idiotcy; in later childhood causing imbecility, or in adolescence causing the various degrees of weak-mindedness, eccentricities, and mental derangement so common at that period. Besides this hereditary faulty development the growth of the nervous system may be arrested by many of the diseases of childhood, such as convulsions, apoplexies, scarlet fever, or by injuries to the head.

II. Overstrain from the pressure of adverse circumstances in the environment is one of the most frequent and powerful causes of insanity. There can be no doubt that our surroundings exercise a most powerful influence either for good or evil upon our mental health. Not less important are they in their relation to the nervous system than are sanitary or insanitary surroundings important as regards the health of the body. There is no more fruitful source of premature mental decay than constant anxiety and worry, and a series of afflictions and losses is often sufficient to unhinge well-regulated nervous constitutions; but as much depends upon the stability of the nervous system as upon the severity of the strain to which it is subjected.

III. Overstrain due to causes acting within the organism.

The most common of these are those due to crises occurring at the different periods of life. At the period of adolescence, which is the transition between childhood on the one hand and manhood or womanhood on the other hand, there is such an influx of new ideas and experiences, that the strain upon weak nervous systems is frequently sufficient to overbalance them. The other periods are those of child-bearing in women, of the climacteric, which is the transition between virility and old age, and the period of senility, or old age itself.

Another important division of internal causes is that of poisons produced within the system, such as those of fevers, the poison of alcohol, and other narcotics imbibed by the individual, and poisons formed during tissue waste and repair. Great physical emaciation and exhaustion, due to failure of the digestive apparatus, or to drains upon the system, or to diseases of the blood, may also act within this category.

